# Crosstalk between mitochondrial biogenesis and mitophagy to maintain mitochondrial homeostasis

**DOI:** 10.1186/s12929-023-00975-7

**Published:** 2023-10-12

**Authors:** Lei Liu, Yanjun Li, Guo Chen, Quan Chen

**Affiliations:** 1 Key Laboratory of Membrane Biology, Institute of Zoology, Chinese Academy of Sciences, Beijing, China; 2 College of Life Sciences, University of Chinese Academy of Sciences, Beijing, China; 3 Institute for Stem Cell and Regenerative Medicine, Beijing, China; 4 Center of Cell Response, State Key Laboratory of Medicinal Chemical Biology, College of Life Sciences, Nankai University, Tianjin, China

**Keywords:** Mitochondrial biogenesis, Mitophagy, Mitophagy receptors, Mitochondrial quality, Aging, Aging-related diseases

## Abstract

Mitochondrial mass and quality are tightly regulated by two essential and opposing mechanisms, mitochondrial biogenesis (mitobiogenesis) and mitophagy, in response to cellular energy needs and other cellular and environmental cues. Great strides have been made to uncover key regulators of these complex processes. Emerging evidence has shown that there exists a tight coordination between mitophagy and mitobiogenesis, and their defects may cause many human diseases. In this review, we will first summarize the recent advances made in the discovery of molecular regulations of mitobiogenesis and mitophagy and then focus on the mechanism and signaling pathways involved in the simultaneous regulation of mitobiogenesis and mitophagy in the response of tissue or cultured cells to energy needs, stress, or pathophysiological conditions. Further studies of the crosstalk of these two opposing processes at the molecular level will provide a better understanding of how the cell maintains optimal cellular fitness and function under physiological and pathophysiological conditions, which holds promise for fighting aging and aging-related diseases.

## **Introduction**

Mitochondria serve as power plants that generate adenosine 5’-triphosphate (ATP) through oxidative phosphorylation (OXPHOS) for the cell. They also contribute to the regulation of calcium homeostasis, intracellular signaling transduction, cellular proteostasis, heme and lipid biosynthesis, reactive oxygen species (ROS) production, and programmed cell death [1–6]. To fulfill such diverse and critical roles in the cell, mitochondria undergo constant fission and fusion cycles to maintain their shape, network, and inheritance. This constant turnover helps maintain their fitness and normal cellular functions. Dysregulation of these critical processes has been causally linked to a myriad of diseases, including metabolic disorders, neurodegenerative diseases, heart and vascular diseases, inflammatory diseases, hematological diseases, and cancers [7–12].

**Fig. 1:**
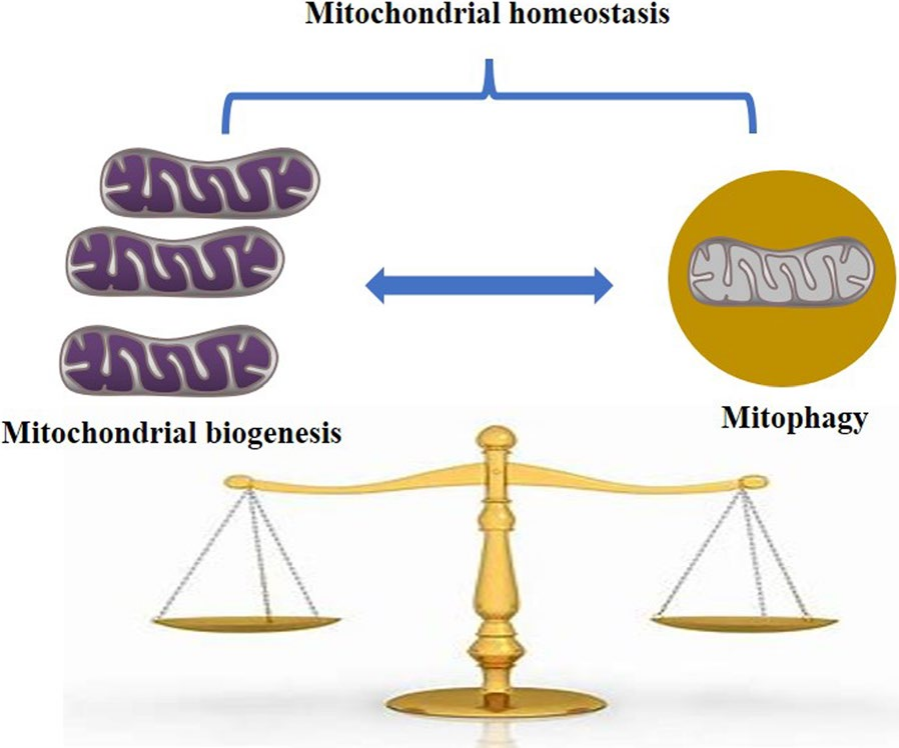
Coordination between mitophagy and mitobiogenesis. Mitophagy and mitobiogenesis are two opposing processes that work together to maintain mitochondrial quality and quantity in response to various physiological and environmental signals

Mitochondria are not generated de novo in eukaryotic cells. The preexisting mitochondria are distributed between the two daughter cells following cell division [13]. Mitochondria have circular DNA (mitochondrial DNA, mtDNA), and mitochondrial biogenesis (mitobiogenesis) involves the replication, transcription, and translation of mtDNA-encoded genes, the interorganelle transport of phospholipids, and the import of nuclear-encoded proteins into mitochondria through the protein translocation machinery of the outer and inner membranes [14]. Mitobiogenesis is a balanced process that also occurs in parallel to the process of removing mitochondria, ensuring that an optimal number of mitochondria persist within the cell. During evolution, cells have gained several strategies to monitor and remove damaged or superfluous mitochondria. One of the major mechanisms of removal is mitophagy, a selective form of autophagy that promotes mitochondrial degradation via the mitolysosomal pathway. In the past decade, both ubiquitin- and receptor-mediated mitophagy pathways have been described [15]. It is less clear how the two opposing processes of mitobiogenesis and mitophagy are coordinated at the molecular level and how they maintain a healthy population of mitochondria (Fig. 1). In this review, we discuss recent advances toward elucidating the molecular mechanisms underlying the coordination of mitobiogenesis and mitophagy. We review the molecular regulation of both mitophagy and mitobiogenesis processes and give special attention to the crosstalk between them that fine tunes the balance of mitochondrial mass and quality.

## Mitobiogenesis

**Fig. 2:**
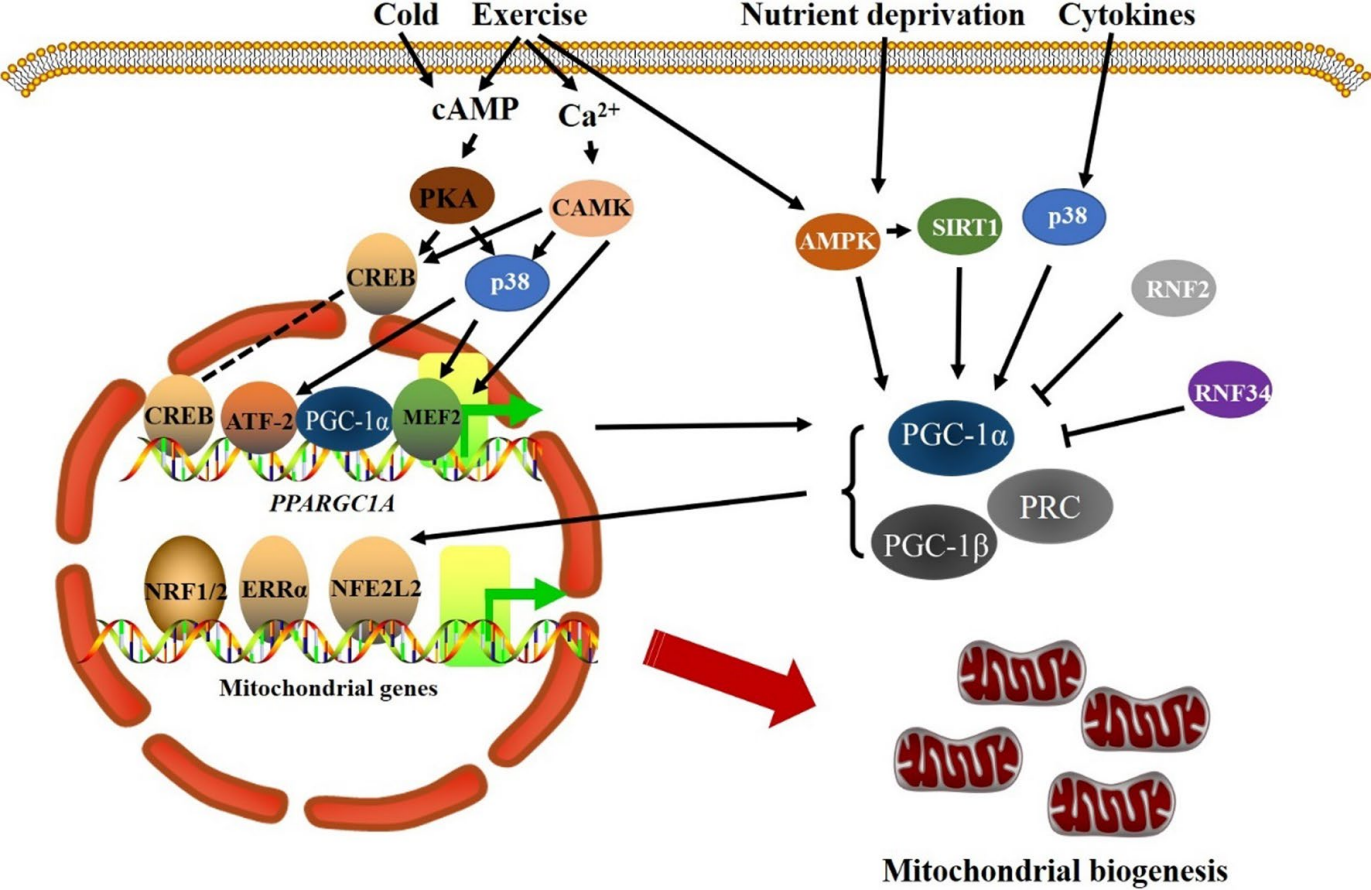
Molecular mechanisms and regulation of mitobiogenesis. The coactivators PGC-1α, PGC-1β, and PRC bind to the transcription factors NFR1/2, ERRα, and NEF2L2 to speed up the transcription of genes involved in mitobiogenesis. MEF2, ATF-2, CREB, and other transcription factors all work together to promote PGC-1α expression, and PGC-1α also improves the transcriptional activity of MEF2 and ATF-2. To trigger mitobiogenesis in response to various physiological and environmental cues, such as cold exposure, exercise, nutritional restriction, and cytokines, these factors are integrated and controlled by several signaling pathways, including the AMPK/SIRT1/PGC1α system, the calcium/CAMK/p38 MAPK/PGC-1α pathway, and the cAMP-PKA pathway

Mitobiogenesis is the process of increasing the number and size of mitochondria in response to higher energy demands and other cellular cues [16]. Given that the majority of mitochondrial proteins are nuclear-encoded, the transcription and translation of nuclear and mitochondrial genes must be tightly coordinated to ensure the creation of new mitochondria [17]. For instance, the assembly of the respiratory chain necessitates the synchronization of the gene expression of the nuclear and mitochondrial genomes [18]. Most mitochondrial precursor proteins generated in the cytoplasm are imported and sorted into mitochondrial subcompartments by the TOM/TIM complex, and the assembly of mitochondrial-encoded and nuclear-encoded subunits faces potential difficulty [18, 19]. Unassembled respiratory chain subunit buildup is linked to elevated ROS generation and mitochondrial proteotoxic stress, both of which have negative effects on the cell [20, 21]. As a result, diverse signaling cascades and transcriptional complexes are needed to ensure appropriate mitobiogenesis, which is regulated by several types of coactivators and transcription factors (Fig. 2) [22]. In addition to transcription factors, mitobiogenesis is also regulated by mitochondrial retrograde signals, such as ATP, ROS and Ca^2+^ [23]. The roles of ATP and ROS in mitobiogenesis are discussed below. Many studies have shown that disruption of mitochondrial membrane potential (Δψm) affects mitochondrial uptake of Ca^2+^, and increased cytosolic Ca^2+^in turn activates various Ca^2+^-dependent kinases and initiates signaling cascades that regulate mitobiogenesis [24].

### Transcriptional regulators of mitobiogenesis

The nuclear transcription factors that were first discovered to play a role in the regulation of mitobiogenesis were nuclear respiratory factors 1 (NRF1) and 2 (NRF2) (Fig. 2) [25, 26]. NRF1 acts as a positive transcriptional regulator because it has a C-terminal transcriptional activation domain (TAD) that binds the promoters of several mitochondrial-related genes [26]. It has now been well documented that NRF1 serves as the master regulator of mitobiogenesis [27]. NRF1 controls the expression of genes encoding respiratory complexes, heme biosynthesis pathway enzymes, components of the mtDNA transcription and replication machinery, and the mitochondrial transport and assembly apparatus [28]. The first mitochondrial gene that was revealed to be upregulated by NRF1 was *cytochrome C*, and a specific NRF1 binding site was discovered in the region upstream of the *cytochrome C* promoter that was necessary for maximal promoter activity [29]. Shortly after, NRF1 was discovered to activate multiple genes involved in respiratory chain complexes [26, 30]. NRFs may also play an integrative role in nuclear-mitochondrial interactions since they increase the expression of key mitochondrial transport machinery components such as TOMM70, TOMM34, and TOMM20 [31]. Additionally, NRF1 activation triggers the transcription of the mitochondrial transcription factor A (TFAM), mitochondrial transcription factor B1 (TFB1M) and B2 (TFB2M) genes, whose products are important regulators involved in the direct control of mtDNA transcription, stimulating mtDNA transcription and its maintenance [22, 32].

NRF2 was initially recognized for its function in the transactivation of the adenovirus E4 gene, also known as GA-binding protein transcription factor (GABP) [33]. It is composed of two separate proteins, NRF2α and NRF2β, which come together to form a tetrameric α2β2 complex. The DNA-binding domain (DBD) or transcriptional activation domain (TAD) is present in NRF2α and NRF2β separately, but both components are required for the generation of a functional complex [34]. NRF2 plays a significant role in the regulation of the transcription of genes involved in the respiratory chain and mitochondrial transcription. Many cytochrome c oxidase (COX) component genes as well as numerous other genes associated with the construction and operation of the respiratory chain have now been unveiled to have functional NRF2 binding sites [28]. Although it has a weaker influence on the activity of the TFAM promoter than NRF1, NRF2 can also bind to the TFAM promoter [32]. In most cases, promoters have both NRF1 and NRF2 binding sites, indicating a mechanism for their coordinated regulation during mitobiogenesis [28].

The orphan nuclear receptor known as estrogen-related receptor α (ERRα) was first identified to control fatty acid oxidation and estrogen signaling. Further studies have demonstrated the crucial function of ERRα in the induction of peroxisome proliferator-activated receptor coactivator 1α (PGC-1α) and 1β (PGC-1β) in mitobiogenesis (Fig. 2) [35, 36]. Many mitochondrial genes include ERRα binding sites, and when ERRα was knocked down, mitobiogenesis driven by PGC-1α or PGC-1β was also reduced [35, 36]. It is noteworthy to state that NRF1 and NRF2 are thought to activate the gene expression of the mitochondrial respiratory chain downstream from the PGC-1α/ERRα axis [37, 38]. Along with controlling mitobiogenesis and function, ERRα can bind to p53, independent of the p53 mutational status. This was found to be essential in preventing ROS generation in cancer cells [39]. In addition, brown adipose tissue (BAT) levels of mitobiogenesis and the expression of genes related to mitochondrial OXPHOS were dependent on ERRα to meet the energy requirements necessary for thermogenesis [40]. Hence, when ERRα is lacking, mice are unable to regulate their body temperatures in response to exposure to cold [40].

By regulating the expression of numerous endogenous antioxidants, nuclear factor erythroid 2-like 2 (NFE2L2) has become a key regulator of antioxidant response element (ARE)-dependent transcription and serves as a master regulator of intracellular redox homeostasis (Fig. 2) [41]. NFE2L2 is typically destined to be ubiquitinated by Kelch-like ECH-associated protein 1 (Keap1), a component of the Cullin 3 (CUL3)-based E3 ubiquitin ligase complex, and is degraded by the ubiquitin‒proteasome pathway within the cytoplasm [42]. When the binding of Keap1 to NFE2L2 is reduced by electrophilic substances or ROS, NFE2L2 is stabilized and translocates to the nucleus, and NFE2L2-dependent cytoprotective genes are activated [43, 44]. It is interesting to note that the NRF1 promoter contains 4 AREs, and overexpression of heme oxygenase-1 (HO-1) activates the NRF1-dependent mitobiogenesis pathway by enhancing the nuclear translocation of NFE2L2 [45]. Moreover, the NFE2L2 activator dimethyl fumarate stimulates mitobiogenesis both in vitro and in vivo [46]. Using NFE2L2 knockout mice, it has been demonstrated that NFE2L2 is essential for mitobiogenesis. In NFE2L2-deficient animals, the esophageal epithelium showed downregulation of 11 mitobiogenesis-related genes, which led to a reduction in mitochondrial mass [47]. In addition, following exercise training, mitochondrial mass in skeletal muscle was also decreased in NFE2L2-defective animals [48].

### Nuclear coactivators

The master regulator of mitobiogenesis, PGC-1α, is a transcriptional coregulator that integrates the activity of many transcription factors, including NRF1, NRF2, and ERRα (Fig. 2) [28]. PGC-1α was originally cloned following yeast two-hybrid screening that utilized peroxisome proliferator-activated receptor γ (PPARγ) as bait. Furthermore, PGC-1α mRNA is dramatically upregulated in mouse BAT and skeletal muscle in response to cold exposure as well as exercise in a cAMP-PKA-CREB-dependent manner, leading to mitobiogenesis and increased respiration [49, 50]. The expression of *UCP1,* a crucial gene involved in thermogenesis, is also enhanced by ectopic expression of *PGC-1α*, which is critical for BAT thermogenesis [49, 51]. Simultaneously, the expression of *PGC-1α *is also significantly induced upon oxidative stress, in turn enhancing the expression of some antioxidant proteins, which can prevent excessive mitochondrial ROS production following mitobiogenesis [52, 53]. Taken together, these findings support the involvement of PGC-1α in mitobiogenesis [49]. Further research has shown that PGC-1α increases the transcriptional activity of NRF1, stimulates the production of NRF1 and NRF2, and increases the expression of TFAM and numerous mitochondrial respiratory chain genes, leading to mitobiogenesis [54]. Additionally, PGC-1α is ubiquitinated and degraded in cancer cells under conditions of protracted glucose shortage that depend on RNF2, and the inhibition of the E3 ligase RNF34 is what causes PGC-1α to stabilize in response to cold exposure in BAT [55, 56]. Many studies have established the dominant involvement of PGC-1α in mitobiogenesis, and the influence of PGC-1α-related mitobiogenesis has also been connected to a number of illnesses, including metabolic disease, cardiomyopathy, neurodegenerative disease, cancer, and kidney disease [57–63].

PGC-1β is a homolog of PGC-1α, sharing a similar tissue distribution and amino acid protein sequence (Fig. 2) [64]. The expression of PGC-1β mRNA, however, is increased during brown fat cell development rather than being triggered by exposure to cold, suggesting that these two proteins have different roles throughout various physiological processes [64]. The fact that PGC-1β promotes transcription by interacting with several transcription factors, including NRF1, ERRα, and ERRβ, suggests that it is also crucial for mitobiogenesis. However, PGC-1α or PGC-1β deficiency had no effect on mitobiogenesis during brown fat differentiation, indicating a complimentary role for these two coactivators in differentiation-induced mitobiogenesis [65]. Indeed, silencing of *PGC-1β *in brown preadipocytes with *PGC-1α* deficiency entirely eliminated the increase in mitochondrial mass and respiration during brown fat formation [65]. Other evidence suggests that PGC-1 coactivators serve an important but complementary role in mitobiogenesis, and this comes from PGC-1α and PGC-1β double deletion animals that died shortly after birth with tiny hearts and a lack of mitobiogenesis in both the heart and BAT [66].

PPARγ-related coactivator 1 (PRC; PPRC1) was first discovered as a transcriptional coactivator. It shares structural similarities and a similar mechanism of action with PGC-1, is widely expressed in mouse tissues, and does not increase in levels in the BAT of mice exposed to cold temperatures (Fig. 2) [67]. PRC interacts with NRF1 and CREB directly and promote NRF1/CREB-dependent gene transcription [67, 68]. Moreover, PRC controls the transcription of NRF2-dependent genes by interacting with host cell factor-1 (HCF-1), and when PRC expression is reduced, TFB2M, COX-II, and cytochrome b are downregulated [69]. PRC expression was rapidly increased by serum stimulation of quiescent cells, which was in contrast to PGC-1α. This suggested that PRC serves complementary roles in controlling mitobiogenesis [67]. The critical role of PRC has also been demonstrated using PRC loss-of-function or knockdown in cultured cells. The expression of respiratory chain proteins and mitobiogenesis were impaired in both cases [67, 70]. Moreover, gene arrays have shown that silencing PRC reduced the expression of almost 50 genes associated with mitochondria, such as respiratory chain subunits, mitochondrial protein import, and assembly factors [70]. In addition, double knockouts of PGC-1α and PGC-1β in skeletal muscle or the adult heart did not result in a loss in mitochondrial mass, which indicated that PRC may play a role in mitobiogenesis in the absence of PGC-1α and PGC-1β [71, 72], a finding that warrants further investigation.

### Signaling pathways that activate mitobiogenesis

The spatiotemporal modulation of mitobiogenesis in response to a variety of stimuli, such as energy need, hormone cues, and environmental stimuli, involves a number of signaling pathways. The coordination of transcription factor networks for mitobiogenesis depends on the integration of different signaling pathways (Fig. 2). Through phosphorylation of PGC-1α and increased PGC-1α stability, AMP-activated protein kinase (AMPK), the main molecular sensor activated by cellular stressors that causes ATP depletion, was found to be a key regulator of mitobiogenesis [73]. While AMPK is active, NAD^+^ levels also rise, which promotes sirtuin 1 (SIRT1) and PGC-1α activity [74]. The AMPK/SIRT1/PGC1α signaling axis is essential for the deacetylation of PGC-1α by SIRT1, which is necessary for adaptive metabolic programming to occur during energy restriction and exercise [74].

As a secondary messenger molecule, calcium and its signaling pathways also control mitobiogenesis in a calcium/calmodulin-dependent protein kinase (CAMK)-dependent manner in skeletal muscle [75]. PGC-1α and mitobiogenesis are specifically promoted in mouse skeletal muscle by constitutively active CAMK expression, and an increase in mitochondria in L6 myotubes is driven by increasing cytosolic Ca^2+^, which could be prevented by using a CAMK inhibitor [75, 76]. PGC-1α expression and activity are controlled by p38 MAPK, and a CAMK inhibitor prevented p38 activation in response to increased cytosolic Ca^2+^, demonstrating that p38 MAPK is a downstream target of CAMK [77–80]. Consequently, exercise-induced mitobiogenesis in skeletal muscle was shown to be mediated through the calcium/CAMK/p38 MAPK/PGC-1 pathway, which is crucial for skeletal muscle adaptation to exercise training.

Another clearly established mechanism, the cAMP-PKA pathway, is crucial for mitobiogenesis in BAT in response to cold exposure. cAMP-PKA phosphorylates CREB at serine (Ser)-133, which activates transcription factors, and directly activates PGC-1 expression via CREB, which is a necessary pathway for mediating glucose homeostasis during fasting [81, 82]. The cAMP-stimulating drugs forskolin and CAMK were unable to induce PGC-1α expression when the cAMP response element (CRE) site was mutated, demonstrating a critical function for CREB in this process. Activating transcription factor 2 (ATF-2), a different CRE-binding protein, is activated by p38 MAPK and is thought to be the primary regulator of PGC-1 gene transcription in brown adipocytes in response to cAMP rather than CREB [77]. Moreover, the expression of PGC-1 is regulated by members of the myocyte enhancer factor 2 (MEF2) family of transcription factors, and PGC-1, CAMK, and p38MAPK all work together to activate MEF2 functions [77, 83].

## Mitophagy

**Fig. 3:**
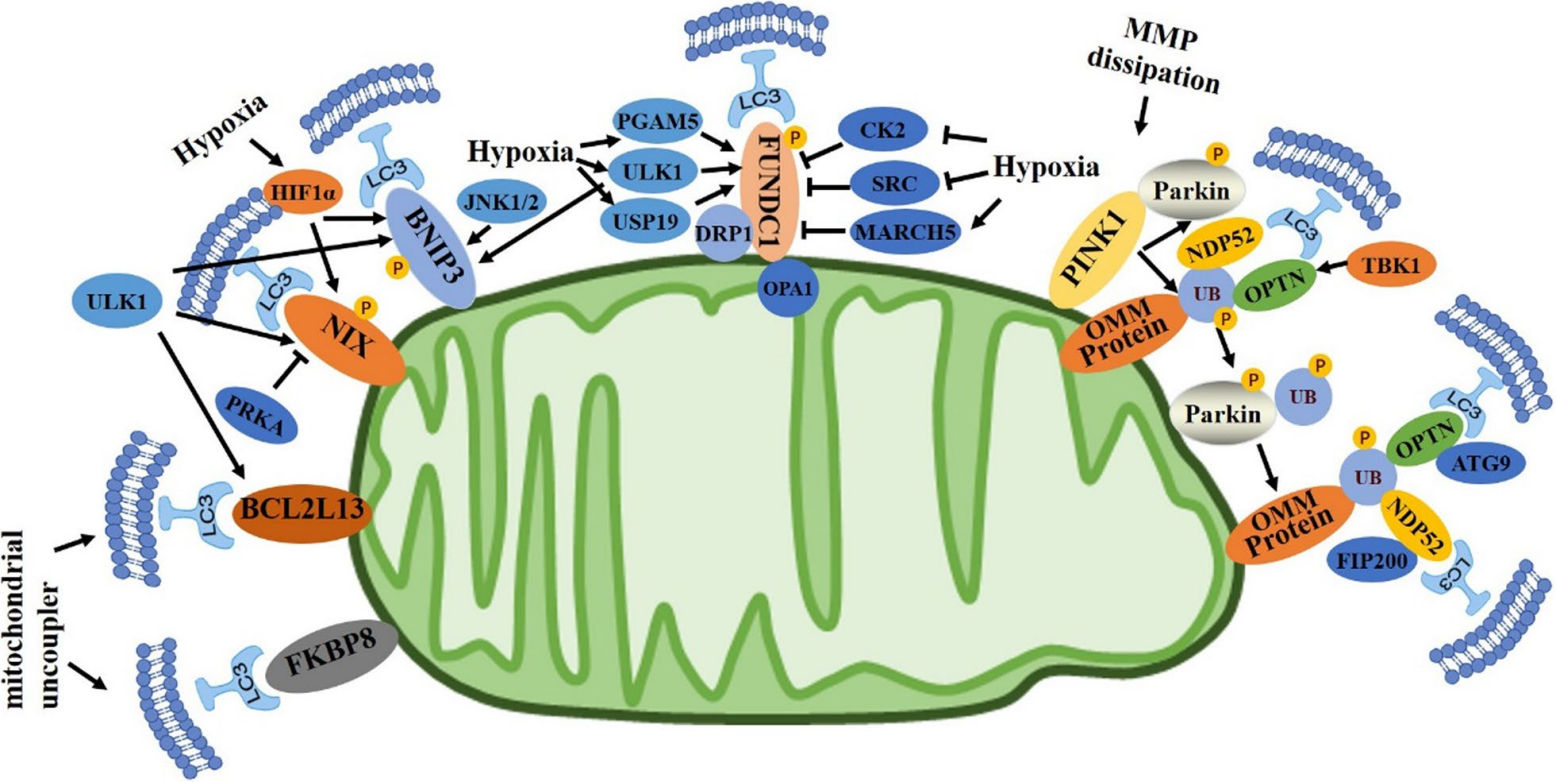
Mechanisms of mitophagy. Mitophagy receptors such as FUNDC1, BNIP3, NIX, BCL2L13, and FKBP8 localize to the OMM and interact with LC3 directly through LIR to induce mitochondrial clearance. The phosphorylation status of these mitophagy receptors and their association with LC3 are influenced by multiple kinases (CK2, SRC, UKL1, JNK1/2, PRKA), phosphatases such as PGAM5, and a variety of stimuli. Together, these influencing factors control the initiation and progression of mitophagy. Dephosphorylation of FUNDC1 promotes its separation from OPA1 and interaction with DRP1, which in turn facilitates mitochondrial fission and the targeted elimination of damaged mitochondria. The E3 ligase MARCH5 and deubiquitinase USP19 also control the protein level of FUNDC1 and mitochondrial fission activity. HIF-1α controls the levels of the proteins BNIP3 and NIX, which are essential for hypoxia-induced mitophagy. Parkin recruitment to damaged mitochondria depends on the stability of PINK1 on the OMM, which occurs as a result of ΔΨm dissipation. Parkin ubiquitinates several OMM proteins, while PINK1 phosphorylates and activates Parkin. Autophagy initiation proteins (ATG9, FIP200) are attracted to damaged mitochondria by several autophagy adaptor proteins, including NDP52 and OPTN, which are brought to the OMM by phosphorylated poly-Ub chains. OPTN is phosphorylated by TBK1, enhancing its interaction with poly-Ub chains and serving as a feed-forward mechanism promoting mitophagy

Macroautophagy, also referred to as autophagy, is a cellular catabolic mechanism that destroys protein aggregates, damaged organelles, and invasive microorganisms through double-membrane vesicles called autophagosomes, which are then degraded in lysosomes. Autophagy is tightly regulated by autophagy-related (Atg) proteins [84–87]. Autophagy is a crucial system for metabolite recycling, minimizing the toxicity caused by damaged organelles or aberrant proteins, and removing pathogenic microbes, which is critical for maintaining cellular fitness [88, 89]. Dysregulation of autophagy has been linked to a variety of illnesses, such as cancer, heart disease, neurological disorders, and metabolic diseases [90–92]. Although autophagy was formerly thought to be a nonselective process, compelling evidence has shown that organelle-specific autophagy is primarily a selective process that is responsible for maintaining the quality and homeostasis of almost all organelles [93, 94]. The term “mitophagy,” which was first used by Lemasters [95], refers to a conserved cellular process that enables the selective destruction of unhealthy or unnecessary mitochondria by autophagy. In most cases, mitophagy is triggered upon oxidative stress and serves as a cellular defense mechanism to remove damaged mitochondria that produce high levels of ROS [96]. As a key cellular quality control mechanism, mitophagy is essential for maintaining the optimal amounts and normality of the functions of mitochondria [97–99]. Mitophagy is also able to promote cell survival by eliminating damaged mitochondria because the release of cytochrome c into the cytosol during mitochondrial damage is associated with the induction of programmed cell death through apoptosis [100, 101]. Furthermore, upregulation of mitophagy by rapamycin, an mTORC1 inhibitor, resulted in a decrease in mtDNA mutant load and rescued cellular bioenergetic function in MELAS or Leber’s hereditary optic neuropathy cell lines, indicating the essential role of mitophagy in reducing mtDNA mutations [102, 103]. As discussed below, mitophagy is primarily regulated by ubiquitin- or mitophagy receptor-dependent mechanisms (Fig. 3) [97, 104].

### Receptor-mediated mitophagy

Mitophagy receptors are proteins that contain the LC3 interaction region (LIR) domain and are located in the mitochondrial outer or inner membrane [15]. In response to various mitochondrial stressors, such as hypoxia, loss of mitochondrial membrane potential, and oxidative stress, mitophagy receptor proteins are engaged in the selective destruction of damaged mitochondria through their interactions with the essential autophagy protein LC3 [15, 105]. In recent years, a number of mitophagy receptors have been discovered, and the function of these proteins in physiological and pathophysiology processes has also been demonstrated [99].

#### FUNDC1

FUNDC1 is a conserved outer mitochondrial membrane (OMM) protein with 3 transmembrane domains, containing an LIR at the N-terminus that is exposed to the cytosol [106] (Fig. 3). FUNDC1 is normally phosphorylated by Src kinase at tyrosine (Tyr)-18 and CK2 at Ser13. Upon mitochondrial stresses, FUNDC1 becomes dephosphorylated by phosphatase PGAM5 or other phosphatases, leading to the enhanced interaction between FUNDC1 and LC3 and subsequent selective mitophagy [106, 107]. Additionally, hypoxia causes Unc-51-like autophagy activating kinase 1 (ULK1) to translocate to the mitochondria and engage with FUNDC1, phosphorylating Ser17 and enhancing the interaction between FUNDC1 and LC3 [108]. Through interaction with and suppression of PGAM5 and phosphorylation of Ser13 in FUNDC1, the essential anti-apoptotic protein BCL-xL negatively regulates FUNDC1-mediated mitophagy [109]. Multimeric PGAM5 dephosphorylates FUNDC1 to trigger mitochondrial fission and the clearance of damaged mitochondria in response to mild cellular stress, and it can also dephosphorylate BCL-xL at Ser62, which was shown to be necessary for anti-apoptotic actions following severe cellular stress [110]. Moreover, when mitophagy is triggered by membrane potential dissipation, PGAM5 is also cleaved by the rhomboid protease PARL, which facilitates the interaction between PGAM5 and FUNDC1 in a Syntaxin-17 (SYN17)-dependent manner [111]. In addition, FUNDC1 directly interacts with dynamic mitochondrial proteins such as the mitochondrial fission protein DRP1 and the inner membrane fusion protein OPA1 [112]. Hypoxia reduces the capacity of FUNDC1 to interact with OPA1 and instead increases its association with DRP1, which causes mitochondrial fragmentation and selective mitochondrial elimination [112, 113]. The ubiquitin-protein ligase MARCH5 and deubiquitinase USP19 also regulate the protein stability of FUNDC1 and its function, respectively [114]. In the early stages of hypoxia, MARCH5 ubiquitinates FUNDC1 at lysine (Lys)-119, which causes FUNDC1 to degrade and prevents the improper elimination of healthy mitochondria [114]. Meanwhile, during hypoxia, a small percentage of FUNDC1 protein is deubiquitinated by USP19 at Lys119, allowing FUNDC1 translocation to MAMs and DRP1-mediated mitochondrial division [115]. In addition, FUNDC1 is acetylated and deacetylated at K104 by Tip60 and HDAC3, which regulates the dimerization of FUNDC1 and thus its ubiquitination by MARCH5 [116].

Given that mitophagy plays a crucial role in maintaining cellular homeostasis, it is not surprising that dysregulation of FUNDC1-mediated mitophagy has emerged as a crucial factor in many physiological processes and pathophysiological conditions, such as cardiac progenitor cell differentiation [117], angiogenesis and neoangiogenesis [118], liver cancer [119], breast cancer [120], metabolic disorders [121–123], cardiovascular diseases [124–130], renal anemia [131], liver injury [132, 133], cerebral ischemia‒reperfusion injury [134], intestinal ischemia/reperfusion injury [135], kidney ischemia/reperfusion injury [136], and chronic obstructive pulmonary disease [137, 138]. Additionally, FUNDC1-mediated mitophagy may inhibit mtDNA release and suppress inflammasome activation and the inflammatory response, which are advantageous mechanisms for cellular homeostasis [119, 123, 131].

#### BNIP3 and NIX

BCL2 and adenovirus E1B 19 kDa interacting protein 3 (BNIP3) was first discovered by using yeast two-hybrid assays that utilized adenovirus E1B 19 kDa protein as bait. BNIP3 was categorized as a dimeric mitochondrion-localized pro-apoptotic protein with a potential BH3 (Bcl-2 homology 3) domain [139, 140]. The homolog of BNIP3 is NIX, which shares considerable sequence homology and pro-apoptotic activity with BNIP3 (Fig. 3) [141]. The promoters of BNIP3 and NIX both include functional HIF-1-responsive elements (HREs) and are elevated by hypoxia, indicating their possible roles in hypoxia-induced apoptosis [142–144]. Mechanistically, expression of either BNIP3 or NIX may result in the opening of the mitochondrial permeability transition pore (mPTP), loss of ΔΨm, and an increase in ROS generation, which leads to both apoptotic and necrotic cell death [145–148].

Subsequent research has demonstrated that autophagy rather than cell death is induced in hypoxic microenvironments in a BNIP3- and NIX-dependent manner [149]. Furthermore, the impaired sequestration of mitochondria into autophagosomes in *Nix* mutant erythroid cells prevents mitochondrial clearance, revealing a crucial function for NIX-mediated mitophagy in the maturation of erythrocytes [150, 151]. Moreover, NIX was shown to be a mitophagy receptor protein with an LIR that specifically interacts with LC3/GABARAP proteins. This association was shown to be required for the clearance of mitochondria during erythrocyte development [152]. Similarly, the BNIP3 homodimer also interacts with LC3 directly through its amino-terminal LIR, acting as an autophagy receptor for the careful degradation of mitochondria and endoplasmic reticulum [153].

The function and stability of BNIP3 and NIX are also regulated by phosphorylation. At the BNIP3 LIR motif, Ser17 and Ser24 phosphorylation promotes its interaction with LC3B and GATE16 [154]. ULK1 was shown to be the kinase that phosphorylates BNIP3 at Ser17, and this association increases the stability of the BNIP3 protein [155]. Moreover, JNK1/2-mediated phosphorylation of BNIP3 at Ser60 and threonine (Thr)-66 regulates protein stability and prevents proteasomal degradation of BNIP3 during hypoxia [156]. Furthermore, phosphorylation of NIX at Ser34 and Ser35 improves its ability to bind with LC3B and recruit autophagosomes to mitochondria [157]. Additionally, cells treated with cadmium to promote mitophagy exhibited increased phosphorylation of Ser81, which was indicated to be essential for NIX-mediated mitophagy [158, 159]. In contrast, PRKA phosphorylates NIX at Ser212, causing NIX translocation to the cytosol and the inhibition of NIX-induced mitophagy and insulin signaling [160].

Several additional novel mitophagy receptors have been discovered recently (Fig. 3). The mammalian homologue of the yeast mitophagy receptor ATG32, BCL-2-like protein 13 (BCL2L13), directly binds to LC3 through the LIR domain to trigger mitophagy [161]. In mammalian cells, FK506 binding protein 8 (FKBP8) serves as an additional outer mitochondrial membrane (OMM) mitophagy receptor that, in an LIR-dependent manner, attracts LC3A to injured mitochondria [162]. Moreover, upon OMM disruption, the inner mitochondrial membrane (IMM) protein PHB2 binds LC3 via LIR, which is essential for Parkin-dependent mitophagy [163].

### Ubiquitin-mediated mitophagy

Parkin is a cytosolic E3 ubiquitin ligase, and mutations in Parkin genes are responsible for the pathogenesis of autosomal recessive parkinsonism (ARP) [164, 165]. A serine/threonine kinase known as PTEN-induced kinase 1 (PINK1), which is located in mitochondria, was also discovered to be connected to ARP in later studies (Fig. 3) [166]. Early research has indicated that the Parkin/PINK1 pathway contributes to mitochondrial dynamic regulation [167–169], as well as maintaining mitochondrial function [170, 171]. The function of Parkin in mitophagy was not fully understood until a key study, carried out in 2008 [172], showed that Parkin facilitates the autophagic clearance of damaged mitochondria by being specifically attracted to mitochondria with diminished ΔΨm after treatment with a mitochondrial uncoupler [172].

A number of subsequent Investigations provided a clearer picture of Parkin/PINK1-driven mitophagy at the molecular level (Fig. 3). Under steady-state conditions, PINK1 is transported to the mitochondrial intermembrane space for proteolytic cleavage and the cleavaged PINK1 is transported to cytosol and degraded by proteaosome [173, 174]. Nevertheless, PINK1 import is prevented by depolarization of the ΔΨm, which stabilizes PINK1 on the OMM and causes Parkin to be recruited to damaged mitochondria [175]. Upon translocation, the E3 activity of Parkin is also increased by the direct phosphorylation of the ubiquitin-like domain at Ser65 by PINK1 [176, 177]. Furthermore, Parkin E3 activity must be fully activated for PINK1 to phosphorylate ubiquitin at Ser65 and release its autoinhibition of catalytic cysteine [178–180]. Autophosphorylation of Ser228 and Ser402 controls the activity of PINK1 and facilitates Parkin recruitment to the mitochondria [181]. After localization on mitochondria, Parkin ubiquitylates and degrades several mitochondrial dynamic-related proteins, such as Miro and mitofusins, causing a pause in mitochondrial trafficking and segregation of injured mitochondria from the healthy mitochondrial network [182–185]. Meanwhile, the autophagy receptor proteins optineurin (OPTN), NDP52 and NBR1 are also recruited to ubiquitinated mitochondria via their ubiquitin binding domain [186, 187]. Later, LC3 is recruited to damaged mitochondria by the omegasome protein double FYVE-containing protein 1 (DFCP1), enabling OPTN-mediated initiation of the autophagosome via its LIR [186]. Further research has shown that the phosphoro-ubiquitin produced by PINK1 attracts NDP52 and OTPN to injured mitochondria in a Parkin-independent manner, acting as an autophagy signal that may be increased by Parkin on mitochondria [188]. Interestingly, TBK1, a key kinase in the innate immune antiviral response, controls the translocation of OPTN to mitochondria, and OPTN but not NDP52 is essential for autophagosome formation after mitochondrial depolarization [189, 190]. The formation of autophagosome membranes around ubiquitinated mitochondria is dependent on the binding of NDP52 and OPTN to the core autophagy proteins FIP200 and ATG9A, respectively [191, 192].

Paternal mitochondria are thought to be eliminated through Parkin-mediated mitophagy in tandem with mitochondrial E3 ubiquitin protein ligase-1 (MUL1), which was demonstrated during the development of the mouse embryo [193]. Parkin and MUL1 appear to have a redundant function in the autophagic clearance of mitochondria upon membrane depolarization, as evidenced by the greatly reduced paternal mitochondrial degradation that is observed upon the depletion of both proteins rather than either one alone [193]. Similar to FUNDC1, Parkin/PINK1-mediated mitophagy reduces inflammation driven by the release of mtDNA, indicating that both receptor- and ubiquitin-mediated mitophagy play important roles in maintaining mitochondrial quality in vivo [194]. However, one study that utilized mito-QC reporter mice revealed that basal levels of mitophagy occurred independently of PINK1 [195].

### Signaling pathways involved in the activation of mitophagy

The signaling mechanisms that initiate mitophagy, in contrast to those associated with mitobiogenesis, have not yet been thoroughly studied. Several pathways have been shown to be involved in the regulation of mitophagy, but the precise underlying molecular mechanisms remain elusive (Fig. 3). According to one study, blockade of p38 or ERK2 effectively inhibited mitophagy but not autophagy triggered by starvation or hypoxia, which indicated that these two kinases are necessary for mitophagy [196]. Additionally, inhibiting the activity of kinases upstream of p38 and ERK2 could also prevent mitophagy, further demonstrating the importance of these signaling pathways in mitophagy [196]. However, Parkin phosphorylation at Ser131 by p38 MAPK reduced its activity and the mitophagy that resulted from the overexpression of A53T-synuclein [197]. Similarly, ERK1/2 activation of ULK1 caused its ubiquitination and degradation, thereby impairing mitophagy [198]. Hence, further studies are required to reach a consensus on precisely how p38 and ERK activated by various stimuli function in the regulation of mitophagy.

Hypoxia-inducible factor (HIF)-1α controls the expression of the mitophagy receptors BNIP3 and NIX, which are necessary in hypoxia-induced mitophagy. When HIF-1α is defective, the reduction in mtDNA and mass caused by extended exposure of MEFs to hypoxia is entirely blocked but could be recovered when BNIP3 was reintroduced [199]. When HIF-1α was knocked out in renal tubules, ischemia/reperfusion (I/R)-induced mitophagy in the kidney was also suppressed, and this mitophagy defect could be corrected by BNIP3 overexpression [200]. HIF-1α also has essential roles in mitophagy induced by the iron chelator DFP, a chemical hypoxia-mimicking agent, wherein mitophagy was abolished by the combined depletion of BNIP3 and NIX [201]. Thus, in both in vivo and in vitro studies, the HIF-1α-BNIP3/NIX signaling pathway was shown to be crucial for the induction of mitophagy by hypoxia.

Direct interaction and phosphorylation of ULK1, an essential regulator of mitobiogenesis, by AMPK was also shown to control mitophagy [202]. AMPK phosphorylates ULK1 at Ser317 and Ser777 under nutrient deprivation conditions, which promotes ULK1 activity and autophagy [203]. Mitophagy deficiency and accumulation of mitochondria occur in the absence of AMPK or ULK1, demonstrating that AMPK-dependent phosphorylation of ULK1 is necessary for mitophagy and mitochondrial homeostasis [204]. Moreover, exercise-induced mitophagy in skeletal muscle is dependent on ULK1 phosphorylation at Ser555, highlighting the significance of AMPK-ULK1 signaling in the induction of mitophagy in vivo [205]. In addition, in response to mitochondrial stress, Parkin is phosphorylated at Ser108 by ULK1 in an AMPK-dependent manner, which is necessary for PINK1-mediated phosphorylation of Parkin at Ser65 and activation of Parkin-mediated mitochondrial clearance [206]. Moreover, ULK1 directly phosphorylates mitophagy receptors such as FUNDC1, BNIP3, NIX, and BCL2L13 to facilitate mitophagy receptor-mediated mitophagy [207].

## The interplay between mitobiogenesis and mitophagy

**Fig. 4:**
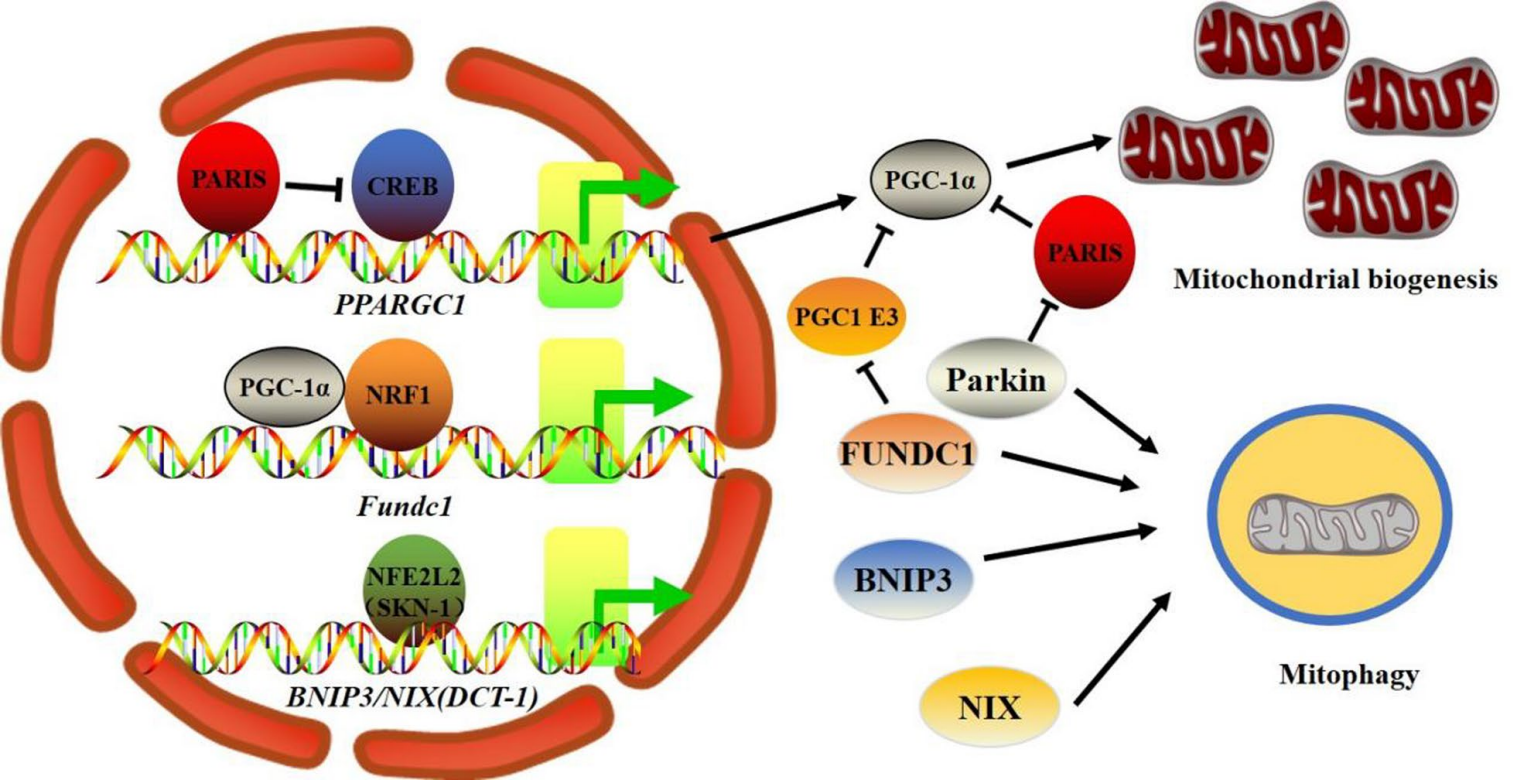
Coordination of mitochondrial biogenesis and mitophagy. PGC-1α is the primary regulator of mitobiogenesis, and in an NRF1-dependent manner, PGC-1α also regulates the expression of the mitophagy receptor *Fundc1*. The NFE2L2 homolog SKN-1 regulates the expression of the BNIP3/NIX homolog DCT-1 in *C. elegans*. In addition, PGC-1α activity can be decreased by mitophagy defects by either controlling its stability (*Fundc1* deficiency) or suppressing its expression in a Parkin-PARIS-CREB pathway-dependent manner

Maintaining optimal mitochondrial content and function requires tightly controlled orchestration of the mitophagy and mitobiogenesis processes. Strong evidence supports the claim that a variety of stimuli can simultaneously elicit mitophagy and mitobiogenesis. In lipopolysaccharide (LPS)-exposed primary mouse lung endothelial cells, MAPK kinase 3 (MKK3) deficiency activated PGC-α-regulated mitobiogenesis and Parkin/PINK1-regulated mitophagy concurrently, resulting in a healthy mitochondrial network and protection against sepsis [208]. Similarly, loss of the putative nutrient-sensing regulator GCN5-like 1 (GCN5L1) resulted in the simultaneous activation of transcription factor EB (TFEB)-mediated mitophagy and PGC-1α-mediated mitobiogenesis, thereby increasing mitochondrial turnover [209]. Moreover, melatonin therapy increased the expression of Parkin/PINK1 and PGC-1α/NRF1 and was beneficial in the prevention of liver fibrosis by enhancing mitobiogenesis and mitophagy [210]. Additionally, thyroid hormone increased the quality of mitochondria by inducing PGC-1α and ULK1 in HepG2 cells and mouse liver tissues, which suggested the potential use of ERRα agonists in metabolic illnesses through enhancing mitochondrial function [211]. Moreover, thyroid hormone increases fatty acid oxidation and mitochondrial respiration in mouse BAT by promoting both mitobiogenesis and MTOR-mediated mitophagy [212]. In mouse renal tissue, the secreted glycoprotein progranulin (PGRN) induces mitobiogenesis and mitophagy through the PGRN-Sirt1-PGC-1α/FoxO1 signaling axis, reducing the effects of excessive hyperglycemia on mitochondrial dysfunction [213]. Myogenic differentiation also requires the coordinated regulation of mitophagy and mitobiogenesis, and the upregulation of p62-mediated mitophagy in the early stages of myogenic differentiation is necessary for subsequent PGC-1α-mediated mitobiogenesis and myogenic differentiation [214]. In *C. elegans*, the processes linking mitobiogenesis and mitophagy have been deciphered (Fig. 4) [215]. The nematode homolog of NFE2L2, SKN-1, is a key regulator of mitobiogenesis and has a role in the control of the expression of the mitophagy receptor DCT-1/NIX in worms [215]. The accumulation of damaged mitochondria caused by DCT-1 or PINK1 knockdown results in the upregulated expression of SKN-1, which is a necessary mechanism involved in counteracting lifetime reduction by mitophagy impairment [215]. In accordance, the cardiac tissues of *Bnip3* and *Nix* double-knockout mice had elevated quantities of mtDNA and mitochondrial proteins [216]. Further work is required to determine whether this axis is evolutionarily conserved and whether NIX is a transcriptional target gene of NFE2L2.

### Parkin/PINK1 in mitobiogenesis

In contrast to BNIP3 and NIX deficiency, Parkin/PINK1 deficiency in neuronal cells suppresses mitobiogenesis, demonstrating that mitophagy coordinates the regulation of mitobiogenesis (Fig. 4). PARIS (ZNF746) is a transcriptional repressor of PGC-1 and NRF1, and the stability of PARIS is controlled by Parkin. As a result, when Parkin is absent, PARIS accumulates, and PGC-1 and NRF1 are downregulated [217]. Conditional depletion of Parkin causes a progressive loss of dopamine neurons in adult animals, which contributes to the pathogenesis of Parkinson’s disease (PD) [217]. As anticipated, Parkin deficiency in the adult mouse ventral midbrain resulted in decreased mitobiogenesis and mitochondrial mass, which could be reversed by PARIS knockdown [218]. In *Drosophila*, the loss of dopamine neurons, mitobiogenesis-related defects, and motor deficits were causally linked to *PINK1* and *Parkin* deficiencies, and these effects were identified to be PARIS dependent. Additionally, phosphorylation of Parkin and PARIS by PINK1 promotes PARIS ubiquitination and proteasomal degradation by Parkin [219]. Moreover, the pathogenesis of PD is significantly influenced by a loss of Parkin-induced mitobiogenesis deficiency rather than mitophagy impairment [220]. Indeed, neurons created from induced pluripotent stem cells (iPSCs) derived from PD patients with *Parkin* mutations have compromised mitobiogenesis pathways [221]. Moreover, *Parkin* knockdown suppresses mtDNA transcription and replication in proliferating cells, indicating that Parkin is directly involved in the control of mitobiogenesis [222]. In SH-SY5Y cells and hepatocellular carcinoma cells, mitochondrial electron transport chain (ETC) function, mitochondrial cristae, and mtDNA levels were decreased when PINK1 was knocked down, indicating a critically important role of PINK1 in mitobiogenesis and in maintaining the activity of the mitochondrial ETC [223, 224]. However, in white adipose tissues (WAT), PARIS protein levels remain unchanged, and PGC-1 is increased when Parkin is absent, indicating that the Parkin-PARIS axis regulates PGC1 in a tissue-specific manner [225]. By increasing PGC-1α protein stability in a mitochondrial superoxide-activated NAD(P)H quinone dehydrogenase 1 (Nqo1)-dependent manner in WAT, deletion of Parkin stimulates mitobiogenesis and protects mice from obesity-causing high-fat diets [225]. When Parkin is absent, the expression of PGC-1 and mitobiogenesis driven by acute exercise in skeletal muscle remain unaffected, indicating that other signaling pathways control mitobiogenesis in this circumstance [226]. It is interesting to note that Parkin overexpression increases PGC-1α expression and mitobiogenesis in the skeletal muscles of aged mice, thereby attenuating fibrosis and oxidative stress caused by aging [227]. Additionally, overexpressing Parkin in *Drosophila* skeletal muscle resulted in an increase in PGC-1α expression and mitochondrial activity [228]. Since Parkin-induced mitophagy decreases mitochondrial mass, it is conceivable that mitobiogenesis is induced to maintain mitochondrial number through upregulation of PGC-1α levels to counterbalance mitophagy when Parkin is overexpressed. Indeed, mitobiogenesis is induced in a TFEB- and PGC1-1α-dependent manner following FCCP-induced mitophagy in SH-SY5Y cells [229]. These findings collectively show that Parkin/PINK1 regulate both mitophagy and mitobiogenesis, and their involvement has significant ramifications for regulating mitochondrial homeostasis and cellular energy metabolism.

### PGC-1α-NRF1-FUNDC1 axis in mitochondrial biogenesis and mitophagy

Investigation of *Fundc1* transcriptional regulation revealed the direct connection between mitobiogenesis and mitophagy (Fig. 4) [230]. Binding of NRF1 to the classic consensus site in the promoter region of *Fundc1* was identified [230, 231]. By using electrophoretic mobility shift assay (EMSA) and chromatin immunoprecipitation (ChIP) analysis, it was determined that NRF1 directly binds to the *Fundc1* promoter. When NRF1 was knocked down, the expression of FUNDC1 was lowered, but this effect was reversed when NRF1 was reintroduced [230]. As mentioned above, the transcriptional activity of NRF1 is stimulated by cofactor PGC-1α, so it is conceived that the expression of *Fundc1* gene  should be regulated by PGC-1α. Indeed, we discovered that PGC-1α and FUNDC1 expression were positively associated in both the BAT tissues of cold-exposed mice and in vitro cultivated adipocytes [230]. The PGC-1α/NRF1 axis is critical for the regulation of *Fundc1* expression, as evidenced by both the direct binding of NRF1 and PGC-1α to the *Fundc1* promoter and the stimulation of FUNDC1 protein production by PGC-1α overexpression [230]. It has been shown that enhanced mitophagy that occurs in skeletal muscles of mice accompanied with acute exercise was attenuated when PGC-1α was deficient and it would be interesting to determine whether FUNDC1 is implicated in this process [232]. Notably, the Eukaryotic Promoter Database indicates a putative NRF1 binding site in the promoter of *Bnip3* and that both NFE2L2 and NRF1 are necessary for the insulin-like growth factor I (IGF-1)-induced activation of the BNIP3 protein [233]. However, it is presently unclear whether NRF1 or other transcription factors involved in mitobiogenesis directly affect the expression of *Bnip3*.

Mouse BAT produces more mitochondria after exposure to cold temperatures, and given that FUNDC1 is a mitophagy receptor, it is conceivable that the mitophagy pathway may also be activated during this process. According to a recent study, the crucial thermogenesis gene uncoupling protein 1 (UCP1) was responsible for elevated mitophagy in BAT from mice following cold exposure [234]. Indeed, mitophagy is activated in cold-exposed BAT, as shown by electron microscopy (EM) visualization of mitochondria inside autophagosomes and the upregulation of autophagy-related proteins [230, 234]. The interaction between FUNDC1 and LC3 was also shown to be amplified in cold-challenged BAT [230]. Brown adipocyte-specific *Fundc1*-deficient mice were used to further demonstrate the significance of FUNDC1 in cold-challenge induction of BAT mitophagy. The mitophagy produced by cold exposure in mouse BAT was dramatically suppressed when FUNDC1 was absent, and this suppression was accompanied by an accumulation of damaged mitochondria, demonstrating that FUNDC1-mediated mitophagy serves as an essential mediator of mitochondrial quality control during mitobiogenesis [230]. Accordingly, *Fundc1*-depleted BAT had decreased mtDNA and mitochondrial function in response to cold exposure, causing poor thermogenesis and cold sensitivity in the animals [230]. Intriguingly, UCP1 induction in BAT by cold exposure was hampered when FUNDC1 was defective due to the lower levels of PGC-1 protein compared to wild-type controls [230]. Given that PGC-1α is a predominant regulator of the expression of UCP1 during cold exposure and that UCP1 plays a crucial role in BAT thermogenesis, it is possible that *Fundc1* loss impairs PGC-1α induction and results in the observed thermogenic abnormalities [230, 235]. The feedback regulation of PGC-1α by *Fundc1* deficiency was also observed in primary cardiomyocytes, and the increased mRNA levels of PGC-1α, NRF1, and TFAM in CK2α-deleted cells were diminished when FUNDC1 was knocked down, suggesting that mitobiogenesis and mitochondrial turnover are accelerated by FUNDC1-mediated mitophagy [236]. PGC-1α and NRF1 directly regulate the expression of *Fundc1*, which in turn provides a direct mechanism for the coordinated regulation of both mitophagy and mitobiogenesis. The rapid clearance of defective mitochondria during mitobiogenesis, which is crucial for maintaining mitochondrial quality and quantity as well as tissue functions, may be caused by activation of PGC-1α/NRF1 control of FUNDC1-dependent mitophagy. It has been shown that inducing mitophagy in a way that is dependent on the small GTPases Rheb and NIX promotes mitochondrial OXPHOS, which in turn stimulates mitochondrial renewal, indicating that mitophagy is committed to the successful renewal of mitochondria [237]. Therefore, by simultaneously activating the two opposing processes of mitophagy and mitobiogenesis, it is possible to speed up mitochondrial turnover and facilitate cells to acquire more healthy mitochondria in response to an increase in energy demand.

Furthermore, when FUNDC1-regulated mitophagy is insufficient, cold-induced mitobiogenesis in BAT is similarly repressed, offering a feedback mechanism to monitor mitobiogenesis and to maintain mitochondrial homeostasis and the ideal number of mitochondria [235]. Our unpublished research has shown that the lack of FUNDC1 increases the interaction between PGC-1α and its E3 ligase while decreasing the protein stability of PGC-1α. However, the details of this retrograde regulation need to be further clarified. Similarly, rosiglitazone-induced mitobiogenesis and upregulation of PGC-1α protein were diminished in differentiated 3T3-L1 adipocytes when *Bnip3* was knocked down, suggesting that efficient mitophagy is required for proper mitobiogenesis [238]. Further evidence supports that the coordination of mitophagy and mitobiogenesis is necessary for supplying energy during differentiation, as indicated by the increased expression of PGC-1α with another mitophagy receptor, BCL2L13, during adipocyte differentiation. Both mitobiogenesis and adipogenic differentiation were suppressed when BCL2L13 was knocked down [239]. The tight coordination of mitobiogenesis and mitophagy allows cells to adjust their mitochondrial content in response to physiological demands, stress, and other intracellular or environmental stimuli, such as cold, hypoxia, and nutrient supply. When mitophagy is impaired, compromised mitochondrial function results in the suppression of mitobiogenesis by decreasing the activity of PGC-1α either by regulating its stability or by repressing its expression to avoid the further accumulation of damaged mitochondria, mitochondrial hyperactivity, and increased production of ROS [217, 236]. In many neurodegenerative diseases, both mitobiogenesis and mitophagy are impaired in neuronal cells, and an imbalance in this regulatory circuit may be responsible for the mitochondria-mediated vicious cycle of oxidative stress [240–243].

### The interplay of mitobiogenesis and mitophagy involved in the metabolism and fitness of organisms during health and disease

To regulate mitochondrial content and quality within cells, there must be a balance between mitobiogenesis and mitophagy. When this balance is disrupted, the functions of organs are hampered, and a variety of disorders are more likely to develop. When *Fundc1* is specifically knocked out in mouse BAT, the mice are incapable of maintaining body temperature in response to short-term or long-term cold exposure [230]. By compromising mitochondrial turnover and quality in response to cold stress, defects in both mitophagy and mitobiogenesis brought on by the loss of FUNDC1 raise the possibility that maintaining the proper balance between these processes is essential in meeting the increased demand for mitochondrial function that arises in some tissues under diverse environmental pressures [230]. The role of autophagy or mitophagy in thermogenesis has also been investigated by using *Atg5* BAT conditional knockout (cKO) mice [212]. Thyroid treatment promoted mitobiogenesis and mitophagy in mice BAT, which raised the body temperature and oxygen consumption rate (OCR) compared to those in euthyroid animals [212]. However, the body temperature of hyperthyroid *Atg5* cKO mice was lower than that of control euthyroid mice, indicating that under healthy conditions, a balance between mitobiogenesis and mitophagy is required to maintain mitochondrial function and thermogenesis [212].

The interaction between mitophagy and mitobiogenesis is crucial for the early postnatal development of the heart and the switch to an exclusively aerobic metabolism [244]. This shift was characterized in cardiac myocytes by elevated mitobiogenesis and Parkin-mediated mitophagy [245]. The genes for mitobiogenesis and mitophagy were downregulated when both Mfn1 and Mfn2 were removed in midgestational and postnatal cardiac myocytes, and the mice went on to develop cardiomyopathy and died within 16 days of birth [244]. Similarly, cardiac *Parkin* deletion or expression of Mfn2 AA (a mutant that hinders Parkin mitochondrial translocation) caused cardiomyopathy and mortality in mice by impairing the replacement of fetal cardiomyocyte mitochondria by mature adult mitochondria [245]. The results of this study showed that the synchronization of mitobiogenesis and mitophagy can promote developmentally programmed mitochondrial turnover, which is essential for maturational metabolic switching to fatty acids in perinatal mouse hearts.

The activity of AMPK, a crucial regulator of mitobiogenesis and mitophagy, is inhibited by poly (ADP ribosyl)ation (PARylation) mediated by PARP1 [246]. The expansion of the lifespan of *Drosophila* occurs when PARP is inhibited in aged flies, which causes AMPK to be activated, increasing mitobiogenesis and PINK1-mediated mitophagy [246]. These findings suggest that increasing mitochondrial turnover may boost mitochondrial health and be a strategy for extending lifespan. [246]. AMPK also plays an important role in the balance between mitophagy and mitobiogenesis in MELAS syndrome, a mitochondrial disorder that is caused mainly by the m.3243 A > G mutation in mitochondrial DNA [247]. Mitobiogenesis works as an AMPK-mediated compensatory mechanism in response to increased mitophagy, and most of the pathophysiological alterations were restored by AMPK activators in MELAS fibroblast lines [247].

On the other hand, a decrease in mitochondrial abundance and function as well as a buildup of defective mitochondria caused by a deficiency in mitobiogenesis and mitophagy have been linked to the regulation of the aging process [248, 249]. Meanwhile, it is widely accepted that the interplay between mitobiogenesis and mitophagy is necessary for the function of neurons and that a failure in this connection is related to a variety of neurodegenerative diseases, including Alzheimer’s disease, Parkinson’s disease, Huntington’s disease, and others [248, 250–253].

## Concluding remarks

It is generally accepted that maintaining cellular and tissue homeostasis requires adequate mitochondrial number and function and that their dysregulation is intimately linked to the onset of many diseases. The regulation of mitochondrial mass and function is governed by two conserved processes, mitobiogenesis and mitophagy. If either one or both processes are disrupted, it results in an accumulation of dysfunctional mitochondria, oxidative stress, cellular aging, and ultimately cell death. Although initially considered two distinct processes, mitophagy and mitobiogenesis are now known to work in concert to maintain the health of mitochondria. More studies are warranted to investigate how cells maintain ideal quantities of mitochondria in response to multiple environmental and developmental signals. It will be interesting to see whether the factors involved in mitobiogenesis also affect the expression of other mitophagy-related proteins, in addition to the previously identified FUNDC1 and BNIP3. Future work will help to determine the mechanisms governing the feedback control of mitophagy dysfunction that leads to a deficiency in mitobiogenesis. As mitochondrial dysfunction is a key characteristic of a plethora of diseases, attempts to elucidate the crosstalk between mitobiogenesis and mitophagy will advance our understanding of the etiology and the development of novel therapeutic approaches for a variety of diseases.

## Data Availability

Not applicable.
